# Behavioral, Transcriptomic, and Neuroinflammatory Effects of Sleep Deprivation: Insights From Separate Acute and Chronic Experimental Paradigms

**DOI:** 10.1002/brb3.71850

**Published:** 2026-10-19

**Authors:** Guohong Cai, Ming Zhang, Suo Zhao, Yifan Lu, Dingding Yang, Jianhong Duan, Jing Wang, Shengxi Wu, Yan Zhao

**Affiliations:** ^1^ Department of Nuclear Medicine, Xijing Hospital Fourth Military Medical University Xi'an China; ^2^ Department of Neurobiology School of Basic Medicine Fourth Military Medical University Xi'an China; ^3^ The 987th Hospital of Joint Logistics Support force of Chinese People's Liberation Army BaoJi China; ^4^ State Key Laboratory of Military Stomatology College of Stomatology Fourth Military Medical University Xi'an China

**Keywords:** **Keywords**: acute sleep deprivation, chronic sleep deprivation, despair‐like behaviors, differentially expressed genes, inflammatory cytokines

## Abstract

**Background:**

Depression is a global health burden associated with sleep disruption and inflammation. While acute sleep deprivation (ASD) can alleviate depressive symptoms, chronic sleep deprivation (CSD) is a risk factor for mood disorders. However, the contrasting mechanisms underlying these opposing outcomes remain poorly understood.

**Methods:**

We integrated behavioral profiling, multi‐region transcriptomics, and multiplex cytokine analysis to separately characterize the effects of ASD in a pain‐depression comorbidity model and CSD in normal mice. Adult male C57BL/6J mice (n = 6 per group) were subjected to either a single 6‐h total sleep deprivation (ASD) or 6‐h daily sleep deprivation for 14 consecutive days (CSD). Behavioral testing included the tail suspension test (TST), open field test (OFT), elevated plus maze (EPM), and sucrose preference test (SPT). Following the interventions, five brain regions—prefrontal cortex (PFC), anterior cingulate cortex (ACC), agranular insular cortex (AI), parietal cortex (PAR), and retrosplenial cortex (RSP)—were dissected. Cytokine levels were quantified using a multiplex panel targeting 15 analytes.

**Results:**

Behavioral tests demonstrated that ASD alleviated despair‐like behaviors induced by spared nerve injury surgery, whereas CSD resulted in anxiety‐ and despair‐like behaviors. The prefrontal cortex (PFC) exhibited prominent transcriptomic responses to both ASD and CSD, with a substantial number of differentially expressed mRNAs. Transcriptome sequencing confirmed stable differential expression of mRNAs across multiple brain regions. Notable genes linked to depression, including Plin4 and Sult1a1, emerged as potential targets for further research. Ingenuity Pathway Analysis of differentially expressed mRNAs in the PFC indicated that IL‐6 and TNF were primarily associated with these changes following ASD. Additionally, inflammatory factors such as BAFF, CHI3L1, IL‐4, IL‐6R alpha, IL‐6, IL‐10, TNFRSF1B, IL‐12 p70, TWEAK, and osteopontin significantly decreased in the ASD group but increased in the CSD group.

**Conclusions:**

This study elucidates that ASD is associated with alleviation of despair‐like behaviors and concurrent suppression of multiple inflammatory factors; however, the causal relationship between these observations requires further experimental validation. These findings open new research avenues for identifying potential depression treatment targets. Future research should explore the functional implications of these transcriptomic changes and their relevance for developing novel antidepressant strategies.

**Significance Statement:**

This study elucidates that ASD is associated with alleviation of despair‐like behaviors and concurrent suppression of multiple inflammatory factors; however, the causal relationship between these observations requires further experimental validation. These findings open new research avenues for identifying potential depression treatment targets. Future research should explore the functional implications of these transcriptomic changes and their relevance for developing novel antidepressant strategies.

## Graphical abstract

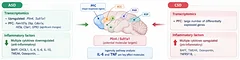

## 1 Introduction

Depression is one of the most prevalent and serious psychiatric disorders globally, closely associated with suicide and various chronic diseases (GBD 2019 Diseases and Injuries Collaborators 2020; Pearce et al. 2022). Sleep deprivation (SD) is recognized as a significant risk factor for mood disorders, including depression (Pires et al. 2016). Therefore, maintaining stable sleep‐wake cycles is crucial for the stability of mood disorders.

Acute Sleep Deprivation (ASD) has been extensively studied and identified as a rapid‐onset therapy for depression (Gardner et al. 2026; Gottlieb et al. 2021; Goldschmied et al. 2023; Boland et al. 2017). While the therapeutic use of ASD may seem counterintuitive, its efficacy is well‐documented. A recent systematic review by Ramirez‐Mahaluf et al. demonstrated that a 36‐h period of SD, when combined with pharmacological treatments, significantly reduces depressive symptoms compared to medications alone (2020). Conversely, long‐term SD strongly correlates with depression development (Okun et al. 2018). Our prior study introduced a chronic sleep deprivation (CSD) model, revealing its ability to induce depression‐ and anxiety‐like behaviors (Cai et al. 2022). The contrasting effects observed between ASD and CSD may be attributed to an incomplete understanding of the mechanisms underlying these two patterns of sleep deprivation.

Furthermore, inflammation, which is implicated in the pathophysiology of depression, may be partially triggered by disruptions in sleep patterns (Irwin 2015; Slavich and Irwin 2014). It is common for individuals with depression to exhibit dysregulation within both innate and adaptive immune systems. Several anti‐inflammatory medications have shown effectiveness in alleviating depressive symptoms in patients with comorbid inflammatory conditions (Cheng et al. 2018). However, to date, no study has systematically compared the changes in inflammation and transcriptome profiles in brain tissue associated with ASD and CSD.

In the present study, here we investigated the behavioral, transcriptomic, and inflammatory consequences of ASD in a neuropathic pain‑induced depression model and those of CSD in normal mice to provide a comprehensive picture of how acute and chronic sleep deprivation, as two separate paradigms, are associated with opposing depression‐related outcomes. Additionally, we analyzed changes in the transcriptome and inflammatory factor levels in key brain regions, especially the prefrontal cortex (PFC), anterior cingulate cortex (ACC), agranular insular area (AI), parasubiculum (PAR), and retrosplenial area (RSP), which were identified in our previous work as particularly responsive to CSD (Cai et al. 2022).

## 2 Materials and Methods

### 2.1 Animals

The study was conducted in male C57BL/6J mice (age, 8–10 weeks). These animals were procured from Fourth Military Medical University. They were kept individually in a room with a temperature of 22–26°C and a 12‐h light/dark cycle. Food and water were provided ad libitum. All experiments were performed in accordance with the Guide for the Care and Use of Laboratory Animals published by the National Institutes of Health (NIH) and were approved by the Institutional Animal Care and Use Committee (IACUC) at the Fourth Military Medical University (Approval No. ACUC‐2021‐1201).

### 2.2 Sleep Deprivation

Mice (*n* = 6 per group) were randomly assigned to either a CSD group or an ASD group. The SD was induced by placing the mice in a separate holding area filled with water (23 ± 2°C) to a depth of 1.5 cm, featuring nine narrow platforms. This setup prevented the mice from sleeping or maintaining a resting position as they attempted to avoid the water (Cai et al. 2022). In the ASD model, mice were kept awake for 6 h. In contrast, the CSD model involved a 6‐hour deprivation period each day for 14 consecutive days, after which the mice were returned to their housing facility. We chose the 6‐hour duration for ASD because it is a well‐established paradigm for inducing rapid antidepressant‐like effects in mice, aligning with the time frame used in our previous publication. For the CSD model, the 6‐hour‐per‐day protocol for 14 days was selected based on our prior work (Cai et al. 2022), which demonstrated that this specific paradigm reliably induces robust anxiety‐ and depression‐like behaviors without causing severe physiological distress or mortality. Following the SD sessions, the mice in the ASD group underwent the tail suspension test (TST), while those in the CSD group were assessed using the open‐field test (OFT), elevated plus maze experiment (EPM), sucrose preference test (SPT), and TST.

#### 2.2.1 ASD paradigm

Sleep deprivation was initiated at Zeitgeber Time (ZT) 0 (lights on, 8:00 AM) and lasted for 6 h until ZT6 (2:00 PM). Behavioral testing (TST) was performed at ZT6–7 (2:00–3:00 p.m.), and tissue collection was completed at ZT7 (3:00 p.m.).

#### 2.2.2 CSD paradigm

Daily sleep deprivation started at ZT0 (8:00 AM) and lasted 6 h until ZT6 (2:00 p.m.) for 14 consecutive days. On day 14, behavioral tests (OFT and EPM) were conducted at ZT6–8 (2:00–4:00 p.m.). On day 15, the SPT was performed from ZT1 to ZT11 (9:00 a.m.–7:00 p.m., 10 h), and the TST was performed at ZT11–12 (7:00–8:00 p.m.). Tissue collection was completed immediately after the TST at ZT12 (8:00 p.m.).

#### 2.2.3 Control animals

All control mice were handled and tested at the same ZT time points as their respective experimental groups (within ±30 min).

All animals were acclimatized to a 12:12 light‐dark cycle (lights on at ZT0, 8:00 a.m.) for at least 7 days before the experiments.

### 2.3 Surgery

Surgery for spared nerve injury (SNI) was conducted as previously described (Decosterd and Woolf 2000). A total of 12 mice were used in this experiment: 6 mice underwent SNI surgery, and 6 mice underwent sham surgery. Under pentobarbital anesthesia, the tibial branches and common peroneal branches of the left sciatic nerve were exposed. The tibial and common peroneal nerves were transected, while the sural nerve was left intact. In the sham group, the nerves were exposed but not transected.

### 2.4 Open Field Test (OFT)

The experimental procedure followed a previously established protocol (Scott et al. 2017). Mice were placed in a white plastic box measuring 40 cm × 40 cm × 40 cm and kept under full‐light conditions (1000 lx). Initially, each mouse was positioned in the center of the box, and its movements were recorded for 5 min. Following the test, the mice were returned to their home cages. An automated analysis system (SMART 3.0, Panlab S.L.U.) was used to measure the time spent in the center of the box, which served as an indicator of anxiety levels.

### 2.5 Elevated Plus Maze Experiment (EPM)

The EPM was conducted following a previously established protocol (Cai et al. 2020). Mice were tested in a four‐arm apparatus, with each arm measuring 50 cm × 5 cm and elevated 1 m above the ground. The maze consisted of two open arms without walls and two closed arms with 15‐cm‐high walls. At the start of the experiment, a mouse was placed in the center of the platform facing one of the open arms. Each session was recorded for 5 min, and an automated analysis system (SMART 3.0, Panlab S.L.U.) was employed to measure the time spent in the open arms as an indicator of anxiety levels.

### 2.6 Tail Suspension Test (TST)

The TST was performed according to a previously documented method (Kim et al. 2017). Mice, which were acoustically and visually isolated, were suspended 50 cm above the ground using adhesive tape applied at one‐third from the tip of their tails. The suspension lasted for 6 min, with the last 4 min specifically analyzed for immobility time, serving as a measure of depression levels.

### 2.7 Sucrose Preference Test (SPT)

The experimental process was as described in a previous study (Yin et al. 2021). Mice were deprived of water and food for 15 h before the preference test (from ZT10 on day 14 to ZT1 on day 15). The electronic SPT device and MDA software were used to conduct preference experiments on day 15. Immediately after deprivation, mice were transferred from home cages into chambers of the apparatus randomly. The duration of the experimental test was 10 h (ZT1–ZT11). Return all mice to their home cages for group housing with adlibitum access to food and water.

### 2.8 MRNA and Differential Expressed Genes (DEGs) Analysis

After anesthetizing the mice, they were immediately decapitated, and the brains were rapidly removed. For transcriptomic analysis, three out of the six mice from each group were randomly selected for mRNA sequencing. Sequencing libraries were prepared using the NEBNext UltraTM RNA Library Prep Kit for Illumina (#E7530L, NEB, USA) according to the manufacturer's instructions, and index codes were added to associate sequences with each sample. RNA concentration in the library was assessed using the Qubit RNA Assay Kit on a Qubit 3.0, while accurate quantification of insert size was performed using the StepOnePlus Real‐Time PCR system.

Differential gene expression analysis between the sleep deprivation (SD) and control groups was conducted using DEGseq v1.18.0, which accommodates non‐biological replicates. This tool is based on the assumption that the number of reads from a gene follows a binomial distribution and employs MA‐plot methodology for analysis. Genes were considered differentially expressed (DEGs) if they met the criteria of *p*‐value ≤ 0.05 and |log2_ratio| ≥ 1.

### 2.9 RNA Extraction and Quantitative Real‐Time Polymerase Chain Reaction (qRT‐PCR)

Total RNAs were purified from various brain regions using TRIzol reagent (Invitrogen 15596026) following the manufacturer's instructions. For quantitative real‐time polymerase chain reaction (qRT‐PCR), RNA was reverse transcribed into complementary DNA (cDNA) using the PrimeScript RT Reagent Kit (Perfect Real Time, Takara RR037A). Reactions were conducted with LightCycler RNA Master SYBR Green I (Roche 03064760001) and quantified using the CFX96 Touch Real‐Time PCR Detection System (Bio‐Rad). Relative gene expression was analyzed with Bio‐Rad CFX Manager and normalized to the internal reference gene GAPDH (Supplementary Table S1).

### 2.10 Cytokine Measurements

LUMINEX multiplex bead array assays were employed to quantitatively measure multiple inflammatory factors simultaneously in different brain regions before and after SD (Ying et al. 2019). Mouse brain tissues were rinsed with PBS, cut into 1–2 mm pieces, and homogenized in PBS using a tissue homogenizer. An equal volume of Cell Lysis Buffer 2 was added, and the tissues were lysed at room temperature for 30 min with gentle agitation. After lysis, debris was removed by centrifugation, and the levels of inflammatory factors in the samples were measured using the Luminex system (Luminex, Austin, TX).

The assay panel included the following targets: B cell activation factor (BAFF), C‐C chemokine ligand 5 (CCL5), Chitinase 3‐like 1 (CHI3L1), interferon‐gamma (IFN‐γ), interleukin‐2 (IL‐2), IL‐4, IL‐6, IL‐10, IL‐12 p70, IL‐27, IL‐6R alpha, matrix metalloproteinase‐3 (MMP‐3), tumor necrosis factor‐alpha (TNF‐α), TNF receptor superfamily member 1B (TNFRSF1B), and tumor necrosis factor‐related weak inducer of apoptosis (TWEAK)/TNFSF12.

Luminex assays were performed according to the manufacturer's protocol. For each mouse in the SD and control groups, samples were analyzed in duplicate, and the average values were used for subsequent analyses.

### 2.11 Statistical Analysis

Data are presented as means ± SEM. Statistical significance was assessed using the unpaired two‐sided *t*‐test for normally distributed data or the Mann–Whitney *U* test for non‐normally distributed data. No statistical methods were used to identify or remove outliers. All data points collected from the experiments were included in the final analyses. Statistical analyses were conducted using GraphPad Prism 6 software, and Venn diagrams were created with DeepVenn. A significance level of *p* < 0.05 was established, with *p*‐values categorized as follows: ***p < 0.001, **p < 0.01, and *p < 0.05.

## 3 Results

### 3.1 ASD Relieved Despair‐Like Behaviors in the SNI Model

Our previous research established the stability of the SNI model in eliciting depressive symptoms (Li et al. 2021). Figure 1A illustrates the protocol for ASD in the SNI surgery group and the sham group. Mice in the SNI group demonstrated significantly decreased mechanical thresholds, as measured by the von Frey test, 60 days post‐surgery, indicating robust mechanical allodynia compared to the sham group (*p* = 0.001) (Figure 1B). Despair‐like behavior was assessed using the TST, where SNI mice spent notably more time immobile than the control group (*p* = 0.006) (Figure 1C), indicating an increase in despair‐like behaviors.

**FIGURE 1:**
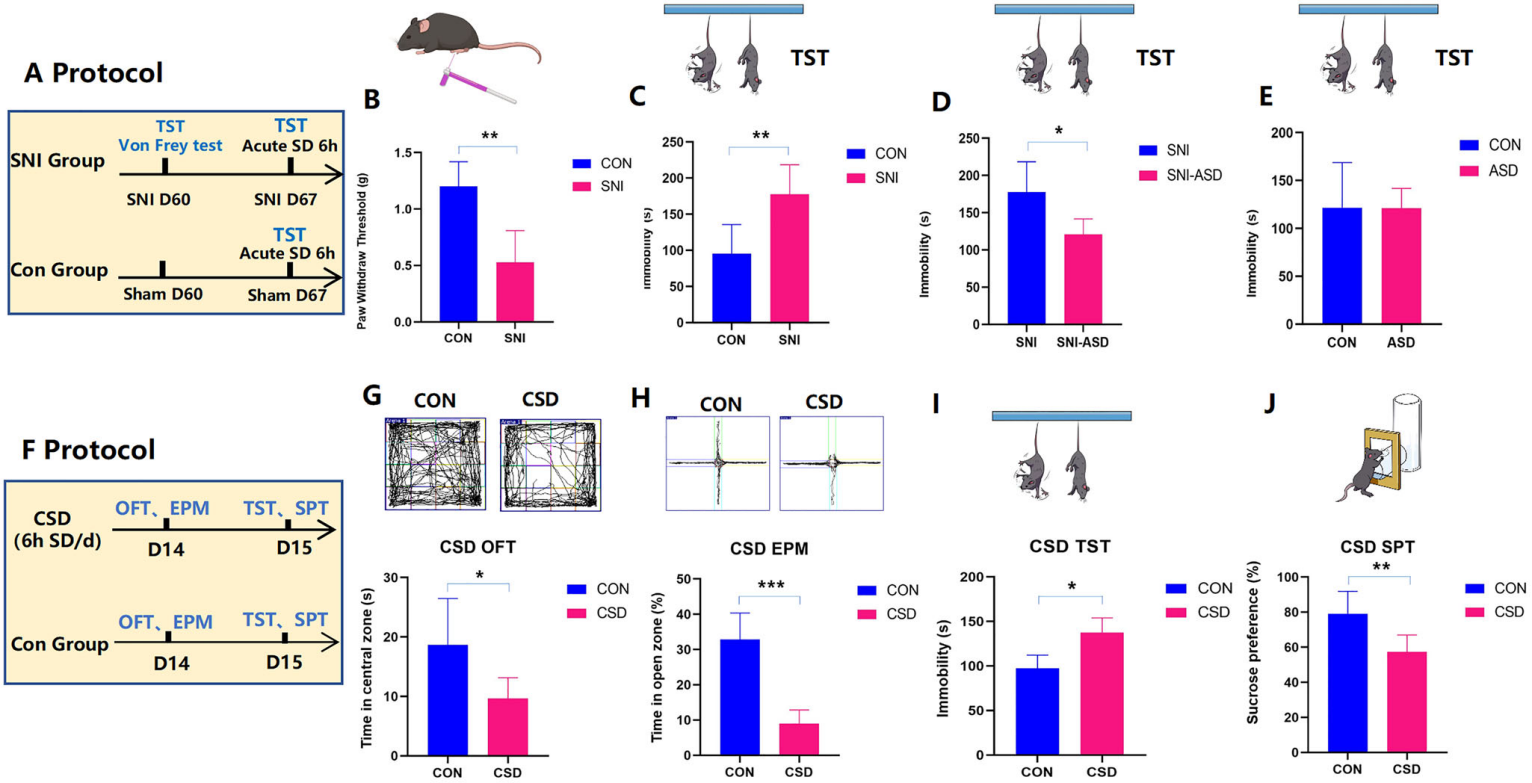
The experimental protocol and behavioral tests. (A) SNI surgery was performed to establish the neuropathic pain model. On day 60 after surgery, mechanical allodynia was measured by Von Frey test. The despair‐like behavior was evaluated by TST. Then ASD was performed on day 67 after surgery. Mice were kept awake for 6 h in ASD model. After ASD, the mice were subjected to TST. (B) The mechanical thresholds of mechanical allodynia in SNI group were significantly decreased compared with sham group after surgery. (C) Compared with the sham group, mice in SNI group spent more time immobile in TST. (D) The mice with SNI spent less time immobile in TST after acute sleep deprivation. (E) The time immobile in TST was similar between acute sleep deprivation and control groups. (F) In the CSD model, sleep deprivation period lasted for 6 h per day and continued for 14 days. The mice were subjected to OFT and EPM on day 14 and to TST and SPT on day 15. (G) Compared with the control group, mice in CSD group spent less time in the center area of the OFT. (H) Mice in CSD group spent less time in the open arms in the EPM test compared with control group. (I) CSD mice spent more time immobile in TST compared with control group. (J) Compared with control group, mice in the CSD showed significantly decreased consumption of sucrose in the SPT. (n = 6 mice per group; two‐tailed *t*‐test). ***P < 0.001, **P < 0.01, and *P < 0.05. Error bars indicate s.e.m. Abbreviations: ASD, acute sleep deprivation; CSD, chronic sleep deprivation; EPM, elevated plus maze experiment; OFT, open‐field test; SNI, spared nerve injury; SPT, sucrose preference test; TST, tail suspension test.

Following the administration of ASD in both SNI and sham mice, the SNI group exhibited reduced immobility time compared to the control group (*p* = 0.013) (Figure 1D), suggesting that ASD effectively alleviates despair‐like behaviors. Conversely, in the sham group, no significant difference in immobility time was observed before and after ASD (*p* = 0.981) (Figure 1E).

### 3.2 CSD Induced Anxiety‐ and Depression‐Like Behaviors in Naïve Mice

In the CSD group, anxiety‐related behaviors were evaluated 14 days after sleep deprivation using the open field test (OFT) and elevated plus maze (EPM) (Figure 1F). Mice in the CSD group spent significantly less time in the center area of the OFT (Figure 1G) and in the open arms of the EPM (Figure 1H) compared to controls, indicating heightened anxiety‐like behaviors. Furthermore, depression‐like behaviors in the CSD mice were assessed using the TST and sucrose preference test (SPT). CSD mice demonstrated increased immobility in the TST (Figure 1I) and reduced sucrose consumption in the SPT (Figure 1J). These findings suggest that CSD mice exhibit depression‐like symptoms, aligning with our earlier work (Cai et al. 2022).

### 3.3 Differentially Expressed mRNAs in Multiple Brain Regions in ASD and CSD Models

To investigate the potential mechanisms underlying the differing states between ASD and CSD, we analyzed the transcriptome in specific brain areas, including the PFC, ACC, AI, PAR, and RSP, in acute and CSD models. These regions were previously identified as the most sensitive to SD in our earlier work (Supplementary Figure S1) (Cai et al. 2022). To determine the functional roles of the differentially expressed (DE) mRNA and their target genes within biological pathways, we conducted gene ontology (GO) analysis for biological functions (Supplementary Figures S2–S5).

The Common DE mRNAs across various brain regions are presented as Venn diagrams, revealing that the AI and PAR exhibited the highest number of shared DE mRNAs (Figure 2A). We validated the expression of these common DE mRNAs in different brain regions using qRT‐PCR. In the ASD group, Sult1a1 significantly upregulated, while Abi3bp was downregulated in the ACC (Figure 2B). In the PFC, the expression of Cbln4 and Crh increased (Figure 2C). The RSP showed significant increases in Plin 4, Sult1a1, and Mfsd2a (Figure 2D). The PAR exhibited an increase in Plin 4, alongside decreases in Lars 2 and Ab i3bp (Figure 2E). The AI group displayed significant increases in Plin4, Sult1a1, Mfsd2a, and Lars2 (Figure 2F).

**FIGURE 2:**
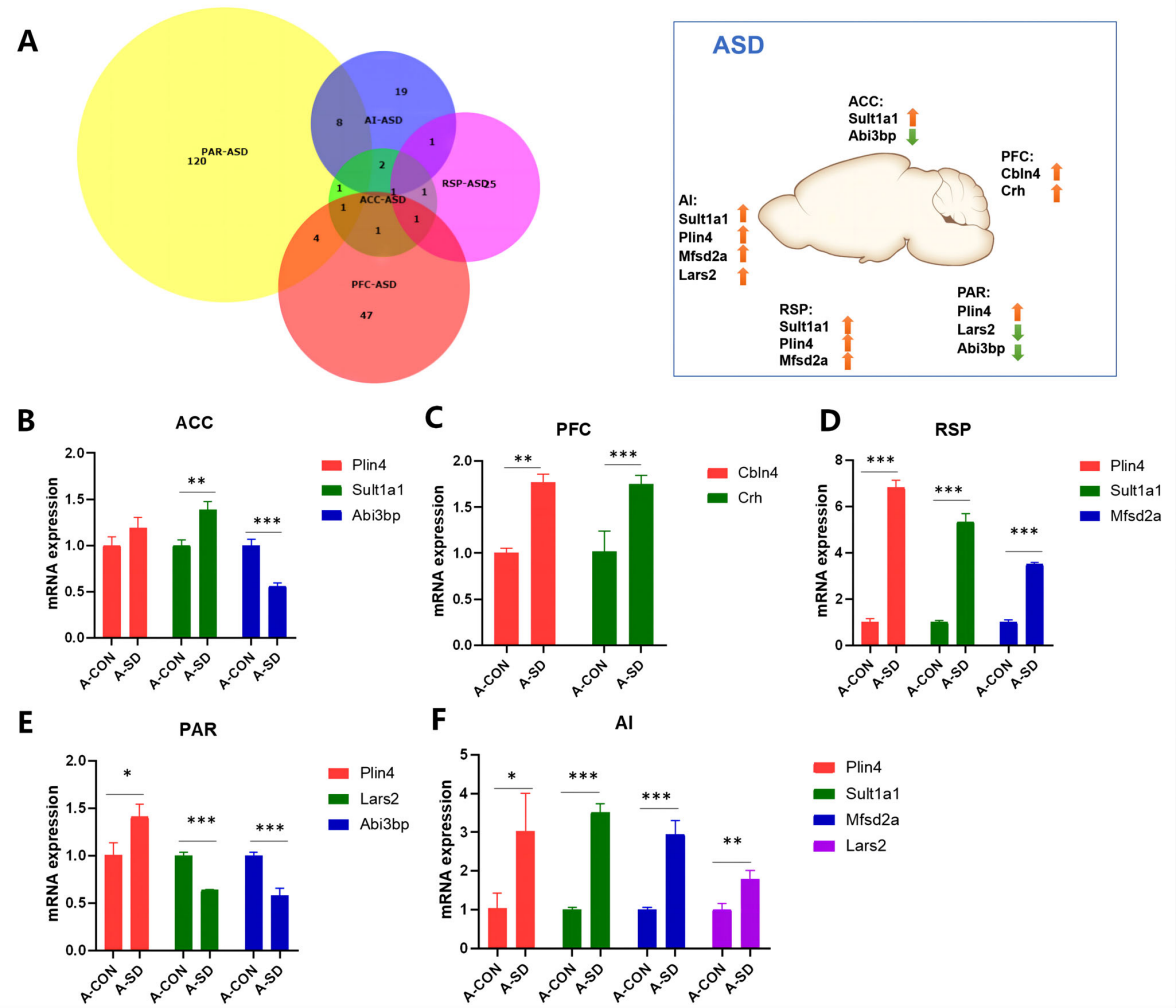
Venn diagrams of the common changed genes of different brain regions in ASD group. AI and PAR had eight common DE genes. Validation of the most common DE mRNAs in different brain regions in ASD group by quantitative real‐time polymerase chain reaction: (B) ACC; (C) PFC; (D) RSP; (E) PAR; (F) AI. (n = 3 per group; two‐tailed *t*‐test). ***P <0.001, **P < 0.01, and *P < 0.05. Error bars indicate s.e.m. Abbreviations: ASD, acute sleep deprivation; PFC, prefrontal cortex; ACC, anterior cingulate cortex; AI, agranular insular area; PAR, parasubiculum; RSP, retrosplenial area.

In the CSD model, the PFC exhibited the highest number of DE mRNAs (Figure 3A), with the AI and RSP showing substantial overlap in DE mRNAs. In the ACC, levels of Gbp4, Car12, Hist1h2be, and mtCo3 significantly decreased in the CSD group (Figure 3B). In contrast, the PFC showed significant increases in Gbp4, Car12, and Etnppl (Figure 3C). The RSP displayed decreased levels of Gbp4 and Cdkn1a, coupled with an increase in Cart12 (Figure 3D). The PAR exhibited increased expression of Hist1h2be, mtCo3, and Tnbs4 (Figure 3E), while the AI displayed elevated levels of Gbp4 and Etnppl (Figure 3F).

**FIGURE 3:**
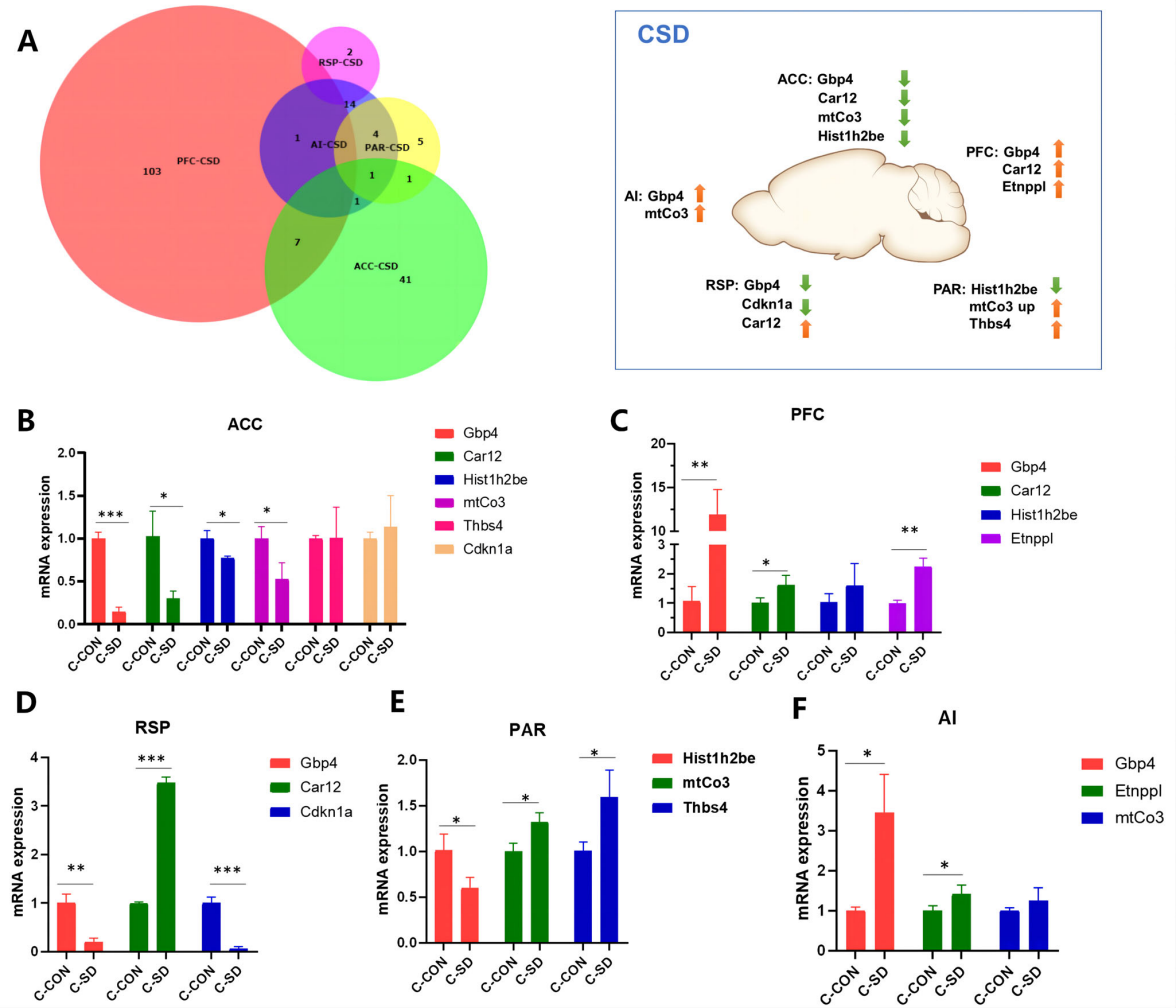
Venn diagrams of the common changed genes of different brain regions in CSD group. AI and RSP had 14 common DE genes. Validation of the most common DE mRNAs in different brain regions in ASD group by quantitative real‐time polymerase chain reaction: (B) ACC; (C) PFC; (D) RSP; (E) PAR; (F) AI. (n = 3 per group; two‐tailed *t*‐test). ***P < 0.001, **P < 0.01, and *P<0.05. Error bars indicate s.e.m. Abbreviations: ACC, anterior cingulate cortex; AI, agranular insular area; CSD, chronic sleep deprivation; PAR, parasubiculum; PFC, prefrontal cortex; RSP, retrosplenial area.

### 3.4 DE mRNAs in the PFC Following ASD: Potential Links to Inflammation

Previous research has established that the prefrontal cortex (PFC) plays a crucial role in the regulation of depression (Dion‐Albert et al. 2022). In the current study, the PFC emerged as the brain region with the highest number of DE mRNAs following SD intervention. To investigate the underlying mechanisms of the effects of ASD, we further analyzed the levels of DE mRNAs in the PFC of the SNI and sham groups. The clustering map of DE mRNAs between the ASD and control groups in sham mice is presented in Figure 4A, indicating 39 upregulated genes and 20 downregulated genes following ASD treatment. Similarly, the clustering map for the SNI mice is depicted in Figure 4B and shows a total of 83 DE mRNAs, consisting of 61 upregulated and 22 downregulated mRNAs after ASD. Remarkably, we identified 22 common DE mRNAs between the sham‐ASD and SNI‐ASD groups, including C5ar1, Gpr3, Cbln4, Hif3a, Cdkn1a, Igfn1, Csrnp1, Mfsd2a, Dbp, Pla2g3, Fam107a, Plin4, Fanced2, Plk5, Gm12840, Sult1a1, Gm13394, Tekt4, Gm42644, Tmem40, Gm7336, and Wnt9b.

**FIGURE 4:**
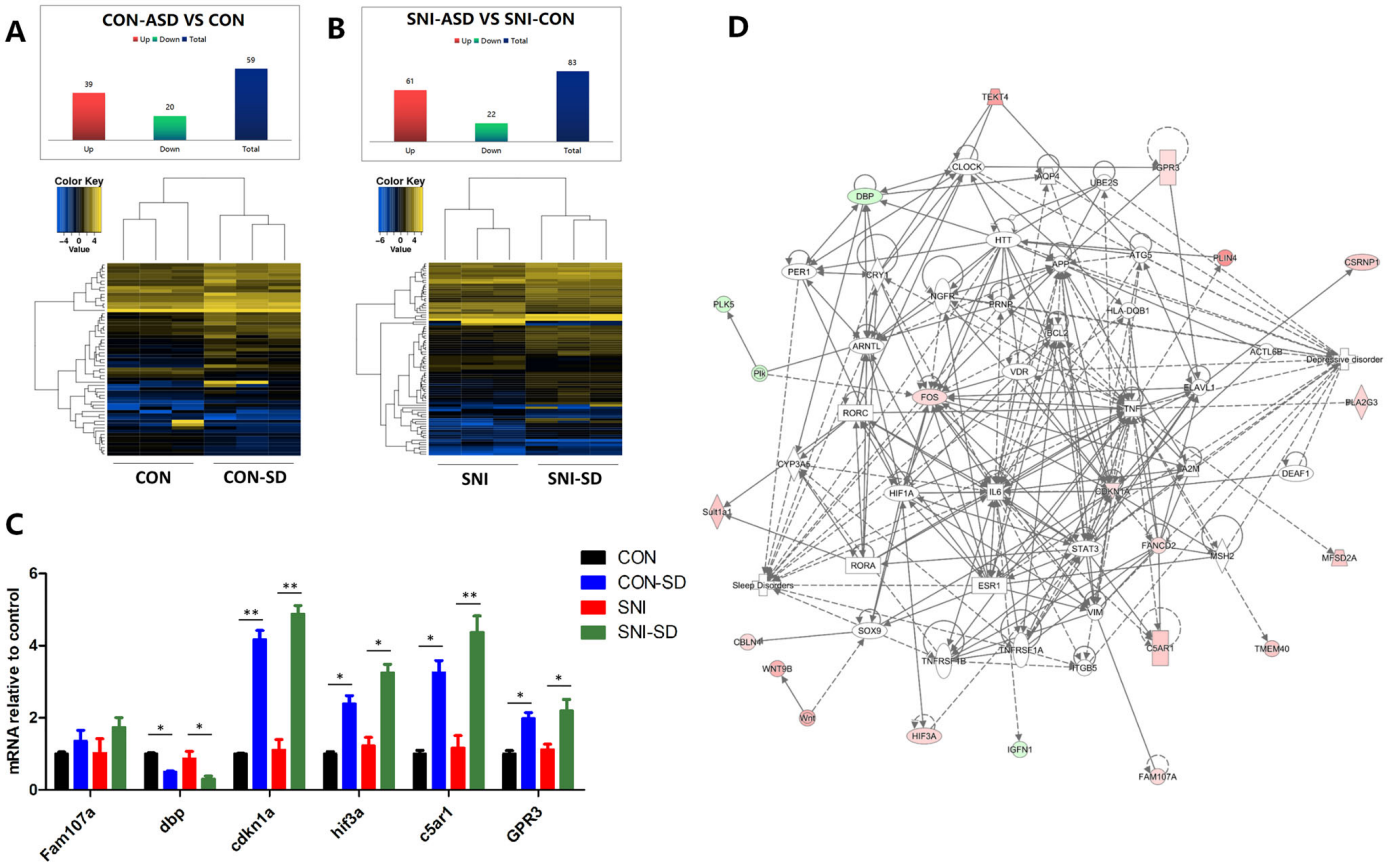
The clustering map and qRT‐PCR of DE mRNAs in PFC. (A) The clustering map of differential expression mRNAs between ASD and control group. (B) The clustering map of DE mRNAs between ASD and control group in SNI mice. (C) Validation of the common DE mRNAs associated with mental disorder including Fam107a, dbp, Ddkn1a, Hif3a, C5ar1 and GPR3 in PFC by qRT‐PCR) (D) The interactome of differentially expressed mRNAs after ASD was generated by Ingenuity Pathway Analysis in PFC. (n = 3 per group; two‐tailed *t*‐test). ***P <0.001, **P < 0.01, *P < 0.05. Error bars indicate s.e.m. Abbreviations: ASD, acute sleep deprivation; DE, differential expression; PFC, prefrontal cortex; qRT‐PCR, quantitative real‐time polymerase chain reaction; SNI, spared nerve injury.

Among these, six genes—*Fam107a*, *Dbp*, *Cdkn1a*, *Hif3a*, *C5ar1*, and *GPR3*—were identified as significantly associated with mental disorders and were subsequently analyzed using qRT‐PCR. The expression levels of most validated mRNAs were consistent with the transcriptomic findings (Figure 4C). Additionally, an interactome of the DE mRNAs was generated through Ingenuity Pathway Analysis, revealing that IL‐6 and TNF were predominantly associated with ASD. This suggests that the antidepressant effects of ASD may be linked to inflammatory processes (Figure 4D). The results of the GO analysis for biological functions are detailed in Supplementary Figure S6.

### 3.5 Effect of ASD and CSD on Inflammatory Factors in Multiple Brain Regions

To further investigate the role of inflammation in sleep deprivation, we analyzed alterations in various inflammatory factors across specific brain regions using a Multi‐Analyte Kit. In the ASD model, we observed a significant decrease in inflammatory factors in most brain regions (Figure 5). Specifically, levels of BAFF, CHI3L1, IL‐4, IL‐6R alpha, IL‐6, IL‐10, TNFRSF1B, and TWEAK were markedly reduced in the PFC.

**FIGURE 5:**
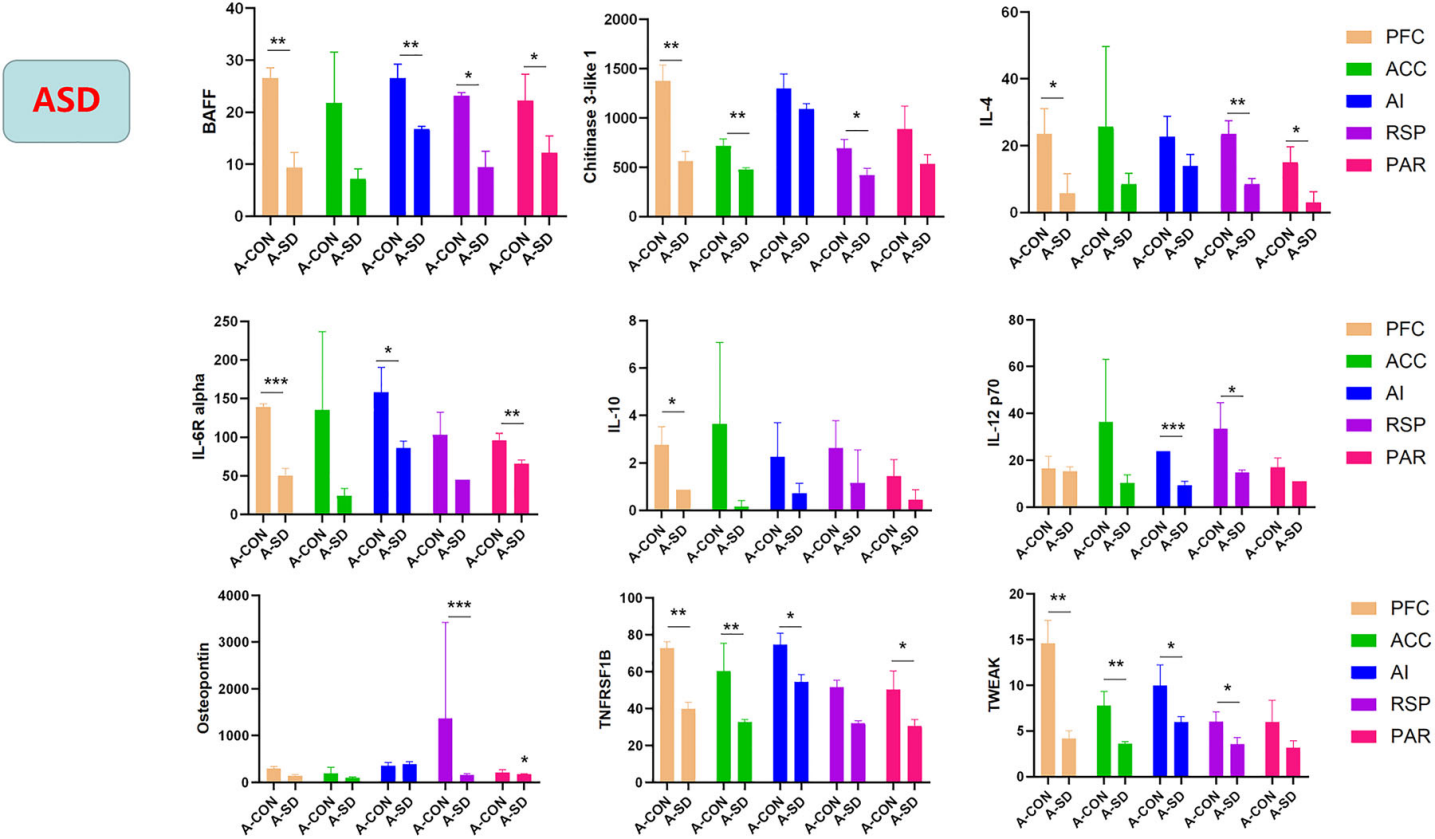
The change of inflammatory molecules between ASD and control groups by LUMINEX multiplex bead array assays. The levels of BAFF, CHI3L1, IL‐4, IL‐6R alpha, IL‐10, IL‐12 p70, Osteopontin, TNFRSF1B and TWEAK were significantly decreased in most brain regions. (*n* = 3 per group; two‐tailed *t*‐test). ***P < 0.001, **P < 0.01, *P < 0.05. Error bars indicate s.e.m. Abbreviations: ASD, acute sleep deprivation; BAFF, B cell activation factor; CHI3L1, Chitinase 3‐like 1; IL, interleukin; TNFRSF1B, tumor necrosis factor receptor superfamily 1B; TWEAK, tumor necrosis factor‐related week inducer of apoptosis.

Similarly, the AI group exhibited decreased levels of BAFF, IL‐6R alpha, IL‐12 p70, TNFRSF1B, and TWEAK. In the RSP, reductions in levels of BAFF, CHI3L1, IL‐4, IL‐12 p70, Osteopontin, and TWEAK were noted. The ACC displayed decreased levels of CHI3L1, TNFRSF1B, and TWEAK, while the PAR exhibited reductions in BAFF, IL‐4, IL‐6R alpha, osteopontin, and TNFRSF1B.

In contrast, the CSD model revealed a significant increases in BAFF, Osteopontin, and TWEAK levels in the PAR, while the PFC displayed an elevation in TNFRSF1B (Figure 6). Conversely, both the AI and RSP groups showed decreased levels of BAFF compared to the control group. These findings highlight a notable distinction in the inflammatory profiles between the ASD and CSD mouse models across the five brain regions studied.

**FIGURE 6:**
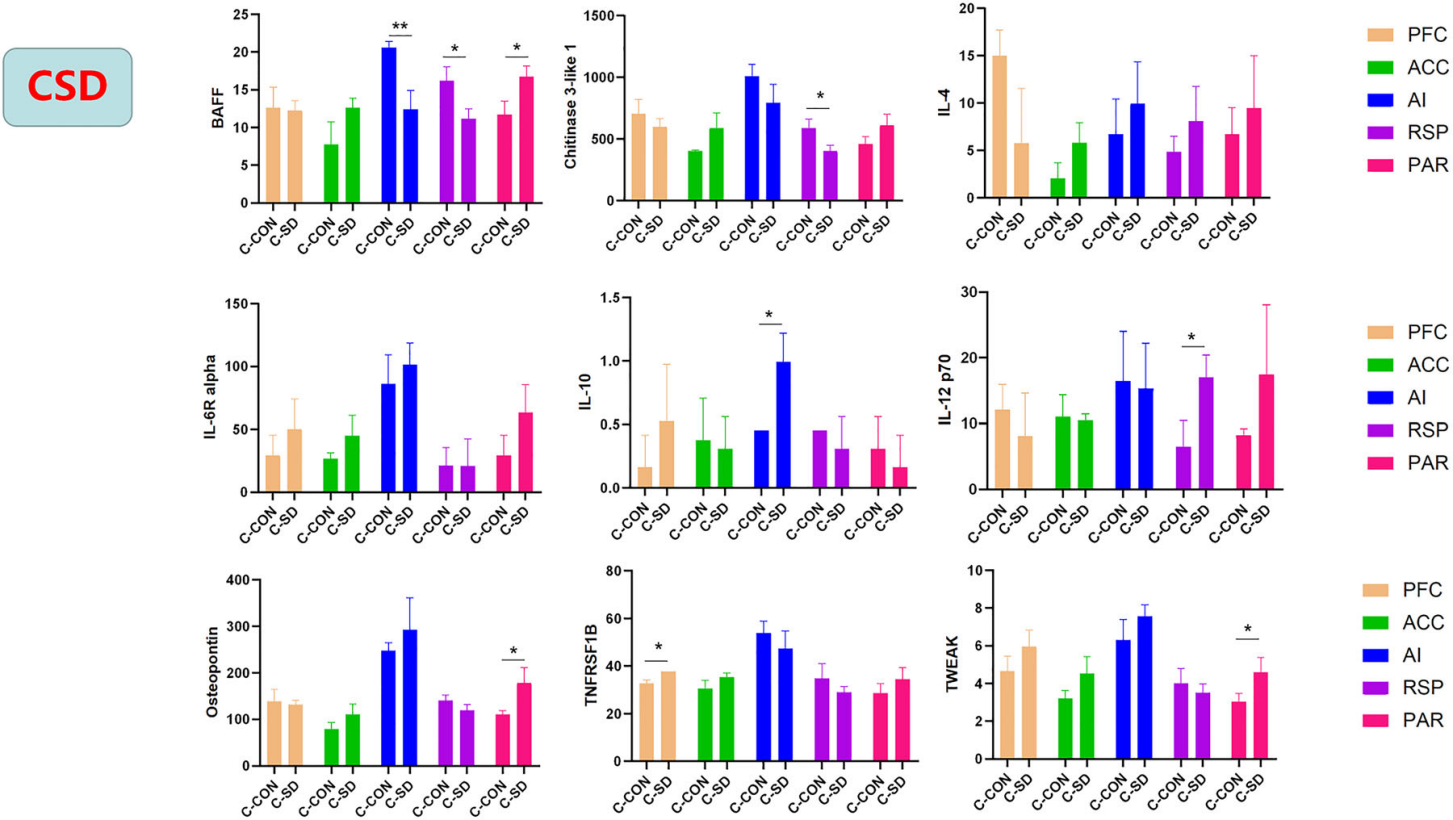
The change of inflammatory molecules between CSD and control groups by LUMINEX multiplex bead array assays. The levels of BAFF and CHI3L1 were decrease in AI and RSP. The levels of IL‐10, IL‐12 p70, Osteopontin, TNFRSF1B and TWEAK were significantly increased in most brain regions. (n = 3 per group; two‐tailed t‐test). ***P < 0.001, **P < 0.01, *P < 0.05. Error bars indicate s.e.m. Abbreviations: AI, agranular insular area; BAFF, B cell activation factor; CHI3L1, Chitinase 3‐like 1; CSD, chronic sleep deprivation; IL, interleukin; RSP, retrosplenial area; TNFRSF1B, tumor necrosis factor receptor superfamily 1B; TWEAK, tumor necrosis factor‐related week inducer of apoptosis.

## 4 Discussion

Chronic sleep deprivation is closely associated with physical diseases, mental disorders, and impaired cognitive function. Interestingly, acute sleep deprivation has been widely utilized as an antidepressant treatment in patients with depression. However, the biological mechanisms underlying both ASD and CSD remain incompletely understood. This study provides a systematic and detailed comparison of the effects of ASD and CSD on the transcriptomes of multiple brain regions. Additionally, we report the impact of different sleep deprivation patterns on inflammatory factors, offering a comprehensive understanding of ASD and CSD characteristics. These findings have significant implications for further research aimed at developing novel treatment strategies for depression.

In our behavioral assessments, we found a significant alleviation of despair‐like behaviors induced by SNI following ASD administration. Unlike conventional antidepressants, which may require several weeks for a complete clinical response, ASD exhibited anti‐depressive effects within hours for approximately 40%–60% of patients (Berman et al. 2000). Previous studies have shown that a notable subset of individuals with major depressive disorder and bipolar disorder experience rapid improvement (within 24 h) with SD therapy. In contrast, CSD is associated with the exacerbation of depressive symptoms and various psychosociological abnormalities. Therefore, investigating the molecular changes that accompany the contrasting changes in ASD and CSD is essential for identifying potential therapeutic targets for depression.

In prior research, we identified a strong association between sleep‐related mood disorders and specific brain regions, including the ACC, AI, PAR, and RSP (Cai et al. 2022). The PFC is also recognized as a critical brain region associated with depression and likely participates in the mechanisms of action of antidepressants (Pizzagalli and Roberts 2022). Consequently, in this study, we conducted transcriptomic analyses of the ACC, AI, PAR, RSP, and PFC in both ASD and CSD models. Overall, the PFC emerged as the brain region most responsive to both ASD and CSD. Analysis of the PFC transcriptome in the ASD model revealed that expression levels of cdkn1a, hif3a, c5ar1, and GPR3 increased following SD, while dbp expression significantly decreased. This trend was consistent between the sham and ASD groups, suggesting a positive influence of the ASD pattern on depression. The DEGs identified, including cdkn1a, hif3a, c5ar1, GPR3, and dbp, may serve as potential treatment targets in future studies.

Furthermore, the analysis of the ASD model revealed a notable upregulation of PLIN4 and Sult1a1 across various brain regions. PLIN4, a member of the lipid droplet (LD)‐associated protein family, plays a crucial role in regulating lipid metabolism, facilitating LD movement, and participating in cellular signaling processes. Han et al. identified elevated PLIN4 levels in the midbrain of mice with Parkinson's disease (PD) induced by 1‐methyl‐4‐phenyl‐1,2,3,6‐tetrahydropyridine (MPTP), suggesting its potential as a biomarker and therapeutic target for PD (Han et al. 2022). Moreover, their findings indicated that knockdown of PLIN4 could reverse mitochondrial damage (Han et al. 2018). Qiao et al. also highlighted PLIN4 as a potential biomarker for depression treatments involving N‐methyl‐d‐aspartic acid receptor antagonists. Notably, mRNA expression levels of Plin4 were significantly higher in striatal samples following ketamine treatment (Qiao et al. 2019).

Sult1a1, or sulfotransferase 1a, specifically catalyzes the sulfonation of dopamine, catecholamines, adrenaline, and noradrenaline. This enzyme is involved in various physiological processes, including cancer progression, hormone regulation, drug metabolism, and neurotransmitter synthesis and metabolism (Thomas and Coughtrie 2003). In line with our findings, the mRNA expression of Sult1a1 is influenced by multiple antidepressant treatments, underscoring its significant role in mood regulation (Conti et al. 2007).

Additionally, sleep has a strong regulatory influence on immune functions (Besedovsky et al. 2012; Cao et al. 2024; Zhang et al. 2024). It is considered to have beneficial pro‐inflammatory effects, which may facilitate the extravasation of T cells and their potential redistribution to the lymph nodes. Sleep occurring post‐vaccination can enhance the subsequent adaptive immune response, functioning similarly to an adjuvant (Besedovsky et al. 2012). In our study, we observed decreased levels of most inflammatory factors in the ASD model. Notably, CHI3L1, also referred to as YKL‐40, showed a significant decline in several brain regions, including the PFC, AI, and RSP. Previous research indicated that elevated plasma levels of CHI3L1 are associated with an increased risk of ischemic stroke, myocardial infarction, and cardiovascular mortality. Increased expression of CHI3L1 was also noted in patients with hypertensive obstructive sleep apnea (Jafari and Mohsenin 2016).

BAFF, a member of the TNF superfamily, plays a critical role in modulating immune responses and regulating apoptosis. In our study, BAFF expression was significantly downregulated in multiple brain regions of the ASD model, including the PFC, AI, and RSP. Consistent with its pleiotropic functions, previous clinical evidence has shown that elevated plasma BAFF levels independently predict an increased risk of incident depressive symptoms over a two‐year period in hemodialysis patients, even after adjusting for conventional inflammatory cytokines such as TNF‐α and APRIL (Lee et al. 2025). Osteopontin (OPN), initially identified in osteoclasts, is integral to bone strength and resistance to fractures. Although it was first characterized in bone tissue, elevated levels of OPN in plasma have been reported in several central nervous system (CNS) diseases, including multiple sclerosis and AD (Yim et al. 2022). In the current study, OPN levels were significantly decreased in the RSP and PAR in the ASD model, while they were increased in the PAR in the CSD model. Interestingly, a recent study by Li et al. indicated that targeting OPN expression might mitigate LPS‐induced depressive‐like behavior (Li et al. 2023).

In addition, TWEAK is a pro‐inflammatory factor that plays a significant role in neuroinflammation by increasing the secretion and expression of pro‐inflammatory cytokines. A growing body of evidence suggests that TWEAK is essential for the pathogenesis of various CNS diseases. Karadag et al. found elevated TWEAL levels in patients with bipolar disorder (BD) compared to healthy individuals (2021). Similarly, Barbosa et al. reported increased plasma levels of TWEAK in BD patients, regardless of their mood state (Barbosa et al. 2017). Concurrently, Wen et al. reported that Fn14, the sole receptor for TWEAK, displayed significantly attenuated neuropsychiatric manifestations in knockout mice (Wen et al. 2013). Corresponding with Wen's findings, we noted that TWEAK levels increased in the PAR of the CSD model but were significantly decreased in multiple brain regions in the ASD model. These results suggest that the reduction of inflammatory factors may contribute to the behavioral effects of ASD, although future functional intervention studies are needed to establish causality.

This study had some limitations. First, this study only used male mice and not female mice; therefore, it is uncertain whether the changes in the transcriptome and inflammatory factors observed in this study are the same in the female mouse model. Second, the transcriptomic changes and alterations in inflammatory factors observed in this study require further experimental validation of their functional roles, including intervention studies to establish causality. Third, it is important to acknowledge that the ASD and CSD experiments were performed in different baseline models (SNI‐induced pathological state vs. naïve mice). Therefore, a direct statistical comparison between the two sleep deprivation patterns was not feasible and is not claimed in this study. Nevertheless, the directionally opposite changes in inflammatory markers and gene expression across brain regions suggest that sleep duration may be a key variable influencing neurobiological outcomes.

In conclusion, our study confirms that ASD is associated with alleviation of despair‐like behaviors and concurrent suppression of multiple inflammatory factors; however, the causal relationship between these observations requires further experimental validation. Key genes such as Plin4, Sult1a1, Dbp, Cdkn1a, Hif3a, C5ar1, and GPR3 may serve as important targets for further research aimed at mimicking the beneficial effects of ASD on depression.

## Author Contributions

**Guohong Cai**: conceptualization, data curation, formal analysis, methodology, funding acquisition, and writing – original draft. **Ming Zhang**: formal analysis, methodology, and writing – original draft. **Suo Zhao**: data curation and formal analysis. **Yifan Lu**: data curation and formal analysis. **Dingding Yang**: formal analysis and methodology. **Jianhong Duan**: formal analysis and writing – original draft. **Shengxi Wu**: formal analysis, funding acquisition, writing – review and editing. **Jing Wang**: conceptualization, formal analysis, writing – review and editing. **Yan Zhao**: conceptualization, data curation, formal analysis, funding acquisition, supervision, writing – review and editing.

## Funding

This work was supported by STI 2030‐Major Projects (Grant No. 2021ZD0201005 to S.W.), the Natural Science Foundation of Shaanxi Province (Grant No. 2025JC‐YBMS‐1018 to Y.Z.), the State Key Laboratory for Manufacturing Systems Engineering (Grant No. sklms2023013 to Y.Z.), the Young Elite Scientists Sponsorship Program by CAST (Grant No. 2022QNRC001 to G.C.), and general support from the China Postdoctoral Science Foundation (Grant No.2023M744298 to M.Z.).

## Ethics Statement

All experiments were performed in accordance with the Guide for the Care and Use of Laboratory Animals published by the National Institutes of Health (NIH) and were approved by the Institutional Animal Care and Use Committee (IACUC) at the Fourth Military Medical University.

## Conflicts of Interest

The authors declare no conflicts of interest.

## Supporting information




**Supplementary Materials**: brb371850‐sup‐0001‐SuppMat.docx

## Data Availability

The datasets generated for this study can be found in the BioProject ID: PRJNA1071510.
