# Supplementary material for: Behavioral, Transcriptomic, and Neuroinflammatory Effects of Sleep Deprivation: Insights From Separate Acute and Chronic Experimental Paradigms
Source: Brain Behav. 2026 Oct 19;16(10):e71850. doi: 10.1002/brb3.71850 (PMC13679767; doi:10.1002/brb3.71850)
Supplement: Supplementary file 1 [file BRB3-16-e71850-s001.docx]

| Supplementary table 1. The following primers were used in quantitative real-time polymerase chain reaction. | | |
| --- | --- | --- |
|  | Forward | Reverse |
| Plin4 | GTGTCCACCAACTCACAGATG | GGACCATTCCTTTTGCAGCAT |
| Sult1a1 | CAACATGGAGCCCTTGCGTAA | ATGAGCACATCATCAGGCCAG |
| Abi3bp | TGGGCTTCAAGTGAGGAGC | CTCTTGTTGCTGTGTGAATCTCT |
| Mfsd2a | AGAAGCAGCAACTGTCCATTT | CTCGGCCCACAAAAAGGATAAT |
| Cbln4 | GCCGTTCTGCTGATTCTAGTG | CTGGGTTCGAGTCGCACAC |
| Crh | CCTCAGCCGGTTCTGATCC | GCGGAAAAAGTTAGCCGCAG |
| Lars2 | CATAGAGAGGAATTTGCACCCTG | GCCAGTCCTGCTTCATAGAGTTT |
| Gbp4 | GGAGAAGCTAACGAAGGAACAA | TTCCACAAGGGAATCACCATTTT |
| Car12 | AACTGGTCCAAGAAGTACCCA | CTATGTAGGTCTATCGGGGACTG |
| Hist1h2be | ACAACGTGCTGAAGCAGAAAGA | GAGTAGCTCTCCTTGCGGC |
| mt-Co3 | ACCGGAAGAAAAAGACAGTGC | GAGGCTCTTGCTCGGTAGG |
| Thbs4 | ACGGCTGAACAAAGCCATC | TTGCTCAGTCTCAGGAGAACC |
| Cdkn1a | CCCCCAATCGCAAGGATTCTT | CTTGGTTCGGTGGGTCTGTC |
| Etnppl | AAAGGCGAACGGTACTTAGACT | CGGCAGCTTTGACCACTTC |
| Fam107a | CAGACCAGAGTACAGAGAGTGG | GTGGTTCATAAGCAGCTCACG |
| Dbp | GGAAACAGCAAGCCCAAAGAA | CAGCGGCGCAAAAAGACTC |
| Hif3a | GAAGTTCACATACTGCGACGA | GTCCAAAGCGTGGATGTATTCAT |
| C5ar1 | ATGGACCCCATAGATAACAGCA | GAGTAGATGATAAGGGCTGCAAC |
| Gpr3 | CCTGCTGGTGGGTAGCTTG | CATCACATAAGTCCGAGTTACCG |

**Supplementary figure 1**. Volcano plot displays differential expression genes in acute and chronic sleep deprivation groups in the brain regions of PFC, ACC, AI, PAR and RSP.

**Abbreviations**: PFC, prefrontal cortex; ACC, anterior cingulate cortex; AI, agranular insular area; PAR, parasubiculum (PAR); RSP, retrosplenial area; ASD, acute sleep deprivation; CSD, chronic sleep deprivation.

**Supplementary figure 2**. The gene ontology analysis of biological functions in ASD and CSD groups in the brain region of RSP.

**Abbreviations:** RSP, retrosplenial area; ASD, acute sleep deprivation; CSD, chronic sleep deprivation.

**Supplementary figure 3**. The gene ontology analysis of biological functions in ASD and CSD groups in the brain region of AI.

**Abbreviations:** AI, agranular insular area; ASD, acute sleep deprivation; CSD, chronic sleep deprivation.

**Supplementary figure 4**. The gene ontology analysis of biological functions in ASD and CSD groups in the brain region of ACC.

**Abbreviations:** ACC, anterior cingulate cortex; ASD, acute sleep deprivation; CSD, chronic sleep deprivation.

**Supplementary figure 5**. The gene ontology analysis of biological functions in ASD and CSD groups in the brain region of PAR.

**Abbreviations:** PAR, parasubiculum; ASD, acute sleep deprivation; CSD, chronic sleep deprivation.

**Supplementary figure 6.** The gene ontology analysis of biological functions in the brain region of PFC. (A) The analysis between ASD and control group in sham mice; (B) the analysis between ASD and control group in SNI mice.

**Abbreviations:** PFC, prefrontal cortex; ASD, acute sleep deprivation; SNI, spared nerve injury.
